# Posterior–Anterior Brain Maturation Reflected in Perceptual, Motor and Cognitive Performance

**DOI:** 10.3389/fpsyg.2017.00674

**Published:** 2017-05-02

**Authors:** Patrícia Gerván, Péter Soltész, Orsolya Filep, Andrea Berencsi, Ilona Kovács

**Affiliations:** ^1^Department of General Psychology, Institute of Psychology, Pázmány Péter Catholic UniversityBudapest, Hungary; ^2^Laboratory for Psychological Research, Pázmány Péter Catholic UniversityBudapest, Hungary; ^3^Bárczi Gusztáv Faculty of Special Education, Institute for Methodology of Special Education and Rehabilitation, Eötvös Loránd UniversityBudapest, Hungary

**Keywords:** contour integration, finger tapping, Navon global–local task, V1, M1, DLPC, brain maturation, gender differences

## Abstract

Based on several postmortem morphometric and *in vivo* imaging studies it has been postulated that brain maturation roughly follows a caudal to rostral direction. In this study, we linked this maturational pattern to psychological function employing a series of well-established behavioral tasks. We addressed three distinct functions and brain regions with a perceptual (contour integration, CI), motor (finger tapping, FT), and executive control (Navon global–local) task. Our purpose was to investigate basic visual integration functions relying on primary visual cortex (V1) in CI; motor coordination function related to primary motor cortex (M1) in FT, and the executive control component, switching, related to the dorsolateral prefrontal region of the brain in the Navon task. 122 volunteer subjects were recruited to participate in this study between the ages of 10 and 20 (females *n* = 63, males *n* = 59). Employing conventional statistical methods, we found that 10 and 12 year olds are performing significantly weaker than 20 year olds in all three tasks. In the CI and Navon global–local tasks, even 14 years old perform poorer than adults. We have also investigated the developmental trajectories by fitting sigmoid curves on our data streams. The analysis of the developmental trajectories of the three tasks showed a posterior to anterior pattern in the emergence of the developmental functions with the earliest development in the visual CI task (V1), followed by motor development in the FT task (M1), and cognitive development as measured in the Navon global–local task (DLPC) being the slowest. Gender difference was also present in FT task showing an earlier maturation for girls in the motor domain.

## Introduction

Early postmortem (e.g., Huttenlocher, 1990; Huttenlocher and Dabholkar, 1997) and positron emission tomography (Chugani et al., 1987; Chugani, 1994) studies on cortical gray matter development have already suggested that maturation does not proceed in a homogenous temporal and topographic sequence, but shows a characteristic posterior to anterior direction. Structural MRI studies strengthened the earlier findings and demonstrated that the caudal to rostral direction is discernible on a large scale (Reiss et al., 1996; Sowell et al., 1999). However, more recent imaging studies revealed that the timing of the regional maturation is far more complex than a canonical back to front progression: the temporal sequence of maturation is more connected to the function served by the specific area rather than its location (e.g., Sowell et al., 2003, 2004, 2007; Gogtay et al., 2004). These studies have shown concordant results on the earliest development of cortical brain regions’ underlying basic sensory (i.e., primary visual area in occipital lobe, primary sensory areas of parietal cortex, primary areas for olfaction and taste in the frontal operculum, etc.) and motor functions (precentral gyrus of the frontal lobe). Furthermore, findings also demonstrated that areas connected to complex pattern processing (inferior, posterior temporal areas) or spatial orientation and attention (inferior parietal regions) mature next, and finally, regions involved in complex executive functions and multimodal integration (orbitofrontal and superior temporal areas) develop well into adolescence.

Similar to gray matter maturation, white matter (WM) also shows a massive change during childhood and adolescence (e.g., Courchesne et al., 2000; Matsuzawa et al., 2001; Sowell et al., 2003). Several cross-sectional structural MRI studies (e.g., Girard et al., 1991; Paus et al., 2001) and a longitudinal study (Thompson et al., 2000) reported that the age-related pattern of myelination also proceeds along a caudal-rostral arc, however, others failed to find these systematic regional differences in WM developmental changes (e.g., Giedd et al., 1999; Courchesne et al., 2000). Diffusion tensor imaging studies showed that the somatosensory pathway matures early in infancy (Qiu et al., 2008), while frontotemporal tracts showed extended maturational trajectories persisting over adolescence (e.g., Lebel et al., 2008; Asato et al., 2010). These results agree with the earlier mentioned anatomical MRI studies on cortical gray matter maturation.

Gender differences in brain maturation are present already in fetal life. Sex steroids and other hormones significantly affect neural development (see e.g., Giedd et al., 1997). Adolescence, with a massive change in hormone levels, results in further sexual dimorphisms in brain development (see e.g., Lenroot et al., 2007; Lenroot and Giedd, 2010). The elevations in luteinizing hormone and inhibin B levels are clear endocrinological markers of the onset of puberty in both genders (Lahlou and Roger, 2004). An increased production of sex steroids in both males and females also accompanies these changes. The onset of hormonal puberty occurs later in boys. Puberty onset determined by inhibin B levels is between the age of 11 and 12 years in boys (Crofton et al., 2002), and between 10.1 and 10.4 years in girls (Addo et al., 2014). These results are in good agreement with imaging studies that have shown differences in the temporal pattern of brain maturation between the genders. An approximately 1–2 years shift between girls and boys has been reported in terms of peaks in gray matter: 8.5 years in females, and 10.5 years in males (Lenroot et al., 2007). Also indicating an earlier maturation for girls, more prominent cortical surface area expansion was found in males compared to females between 8 and 14 years of age (Koolschijn and Crone, 2013). There are gender differences in WM development in terms of timing and volumetric changes as well. During childhood and adolescence females have an overall earlier maturation of WM tracts than males (Asato et al., 2010), while boys show a far more prominent WM volumetric increase than girls (De Bellis et al., 2001; Lenroot et al., 2007). Current MRI studies seem to validate the view that WM volume increase during the teenage years is associated with testosterone levels and androgen receptor genes in adolescent boys (Perrin et al., 2008; Paus et al., 2010), and luteinizing hormone levels in both genders (Peper et al., 2008).

**FIGURE 1:**
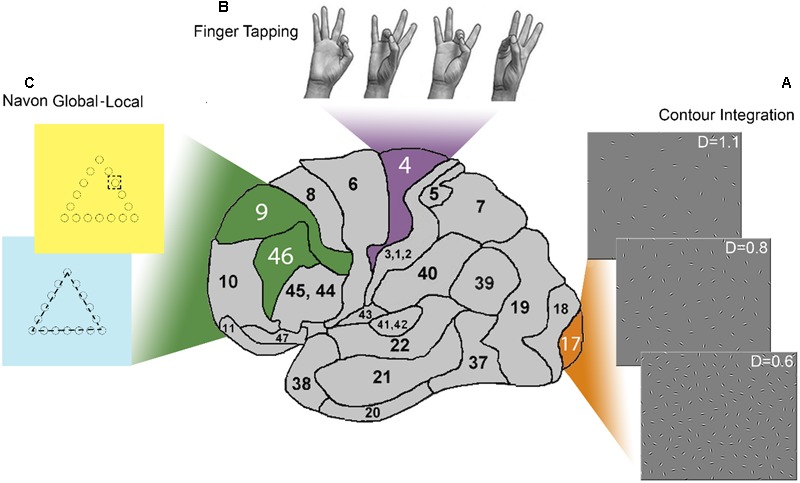
**Summary of the paradigms and related brain regions.** This side view of the human brain shows the three Brodmann areas (BA) addressed by the tasks used in this study. **(A)** The panel shows stimuli at three levels of difficulty in the Contour integration (CI) task. These stimuli are addressing long-range connections in the primary visual cortex (V1; BA 17). The collinear chain of Gabor patches forming a horizontally placed egg-shape is hidden in the background of randomly positioned and oriented noise elements. Relative noise density varied through six difficulty levels. **(B)** Four-element movement-sequence in the Finger tapping (FT) task addressing long-range connectivity of the primary motor cortex (M1; BA 4). Subjects were instructed to touch four other fingers with the non-dominant thumb in a given order which was index – ring – middle –little finger. Participants had to carry out the sequence repetitively, as fast and as correctly as they could. **(C)** The Navon global–local (Navon GL) task consisted of hierarchical stimuli of geometric shapes where the lines of a larger, “global” shape are composed of much smaller, “local” figures (cc. 10th the size of the large ones). Participants were instructed to identify the shape, either at the global or the local level, depending on the color of the background, which was yellow or blue, respectively. This task requires the use of a set of executive control components such as switching and updating which have neural correlates in the dorsolateral prefrontal cortex (BA 46. BA9).

Our purpose was to investigate whether the posterior–anterior wave of cortical structural changes, possibly determined by pubertal hormones, can be matched to a similar wave of improvement in behavioral function. The issue of matching whole-brain cortical structure to function is a complex one, especially in the developmental domain. However, an approximation with probes at strategic points both in terms of structure and function, and in terms of developmental time might be a good start. To this end, we selected well-established behavioral tasks that are believed to be localized differently along the sagittal axis of the brain. We use these tasks to probe how anatomical maturity might be linked to developmental trajectories of different functions (see **Figure 1**).

### Behavioral Tasks as Probes of Posterior–Anterior Brain Maturation

#### Contour Integration Task

Stimuli consist of a collinear chain of Gabor patches embedded in the background of randomly positioned and oriented noise elements (see e.g., **Figure 1A**). This paradigm has been developed to test the long-range intrinsic cortical connections in the primary visual cortex (Field et al., 1993; Kovács and Julesz, 1993). This paradigm has been extensively studied in the last several decades in the field of visual integration, and it has a well-established account on the underlying neural mechanisms. Neurophysiological studies described a correlation between the responses of neurons in V1 and the perceptual saliency of contours (Li et al., 2006), supporting the idea that V1 has a cardinal role in contour integration. Optical imaging of contextual interactions in monkeys (Kinoshita et al., 2009), human neuropsychological (Giersch et al., 2000), and fMRI (Altmann et al., 2003; Kourtzi et al., 2003) studies also indicate the relevance of low-level visual areas in detecting and integrating the contour elements embedded in noise.

#### Finger Tapping Task

Finger tapping is a motor coordination paradigm. It has many variations in terms of the complexity of the tapping task and a ‘pacing’ stimulus. Here we used a self-paced version where participants have to touch the thumb with the other fingers in a given four-element sequence (see **Figure 1B**). Motor function of distal arm and hand movements is controlled by four distinct regions in the frontal lobes: the primary motor cortex (M1), supplementary motor area, the lateral premotor cortex and the cingulate motor area (Todorov, 2000; Lemon, 2008). Direct input to the spinal cord from these areas is shown to be necessary for manual dexterity in primates (Dum and Strick, 2004, 2005). The majority of cortical motor neurons that give direct input to lower motor neurons in the spinal cord originate in M1 (Seo and Jang, 2013), ensuring fast and selective activation of hand muscles. Several studies combined FT with imaging techniques, TMS or electrophysiology leading to increasing amount of evidence of practice driven changes in M1 (Karni et al., 1995, 1998; Muellbacher et al., 2002; Zhu et al., 2010). Changes occurred in the contralateral primary motor cortex during simple, self-paced movements.

#### Navon Global–Local Task

In the Navon global–local task (Miyake et al., 2000), a geometric figure (a greater figure is composed of several smaller figures; see **Figure 1C**) is presented to the subjects, and they are requested to respond either to the “global,” or to the “local” stimuli depending on a visual cue (Navon, 1977). This cue-based switching requires interpreting a display of symbols, selectively attending to certain features and ignoring others, and remembering and applying a complex set of rules. Shifting, or in other terms, switching is an executive control component identified by Miyake et al. (2000). It is assumed to be a component of any goal-directed behavior (Miller and Cohen, 2001). Neuroimaging studies have linked the performance on the Navon global–local task mainly to prefrontal, parietal and subcortical areas, and especially to the bilateral anterior cingulate cortex (ACC, BA 32), the dorsolateral prefrontal cortex (DLPFC, BA 9 and 46), the medial regions of the inferior frontal gyrus (BA 45/47) extending into the insula, and the inferior parietal lobule (IPL, BA 7 and 40) (e.g., Hedden and Gabrieli, 2010). Switching has generally been related to dorsolateral prefrontal cortex (e.g., MacDonald et al., 2000; Monsell, 2003). This is consistent with neuropsychological findings demonstrating that patients with left frontal damage perform poorly in switching between attributes (Rogers et al., 1998; Keele and Rafal, 2000). In a task-switching version of the Stroop test, the left DLPF seemed to contribute to the maintenance of task demands in switching, while ACC activation was related to performance monitoring (MacDonald et al., 2000). The role of parietal regions in the Navon global–local task was investigated in repetitive transcranial magnetic stimulation (Qin and Han, 2007) and transcranial direct current stimulation (Bardi et al., 2013) studies. The results show that the parietal areas are involved in attaining the level of stimulus representation and not directly in the process of switching.

## Materials and Methods

### Subjects

One hundred and twenty-two volunteer subjects participated in this study (female *n* = 63, male = 59). Subjects were voluntarily recruited via the Internet by publicizing and advertising our research. All participants were healthy, right-handed individuals with normal or corrected to normal vision. Participants had no history of psychiatric or neurological illnesses, including ADHD, and were free of any medication. Adult participants or the legal guardians of subjects under the age of 18 were provided with written information, and were asked for consent before participating in the study. Subjects participated in two experimental sessions at the Developmental Neuroscience Laboratory of the Institute of Psychology at PPCU, and were paid for their attendance.

The sample included subjects between the ages of 10 and 20, in 6 age groups. Five subjects were excluded based on their inadequate performance on at least one of the subtests. Additional subjects were recruited subsequently. **Table 1** shows the demographics of the sample.

**Table 1:** Demographic data of the participants.

	*N*	Mean age	*SD*	Male to female ratio
10 years	21	10.57	0.31	11:10
12 years	22	12.53	0.30	10:12
14 years	18	14.52	0.31	8:10
16 years	19	16.59	0.25	9:10
18 years	20	18.57	0.28	10:10
20 years	22	20.61	0.32	11:11

The study was approved by the Ethical Committee of the Institute of Psychology at PPCU.

### Procedure

#### Contour Integration Task

Stimuli were composed of a collinear chain of Gabor elements forming a horizontally placed egg shape on a background of randomly positioned and oriented Gabor patches (see **Figure 1A**). The relative noise density (D) was varied throughout six difficulty levels. D is defined as the ratio of average noise spacing over contour spacing (see Kovács and Julesz, 1993). In our study, D ranged between 1.1 and 0.6, and was varied with a step size of 0.1. Blocks of images were presented in an increasing order of difficulty, starting with the easiest (*D* = 1.1) level, and followed by more difficult levels (up to *D* = 0.6), in a two-alternative forced-choice procedure. The CI task was presented using a HP ProBook 450 G3 laptop computer with a 15.6 inch monitor, and was programmed in Delphi language. Stimulus duration was 2000 ms, with a fixation cross between stimuli (0.5 s, or until the subject responded). The task was to indicate which side of the screen the narrower part of the egg was pointing to by pressing one of two assigned buttons on the keyboard. Subjects were tested binocularly and were seated at about 0.4 m away from the monitor in a normally lit testing room. The size of the stimulus field was 19.93 × 26.57 degrees of visual angle.

#### Finger Tapping Task

Participants were asked to touch the thumb with the other fingers of the non-dominant hand (see **Figure 1B**) in a predetermined order, and as fast and as correctly as possible. 160 repetitions of a four-element-sequence (index-ring-middle-little finger) were distributed into 10 practice blocks with rests between them (the length of the rest was controlled by the subjects). Data acquisition was performed by a custom made data glove. It consisted of metal rings placed on each fingertip that were connected to a PC with a Java-based data acquisition software that detected exact timing and order of finger taps.

Taking the speed/accuracy trade-off into account, motor performance was monitored both in terms of speed and error rate. Performance rate was calculated by multiplying the time between finger taps (speed) with the ratio of the number of finger taps in incorrect sequences compared to all sequences (error rate). In order to eliminate the effect of different corticospinal tract myelination levels (due to e.g., age) on speed, participants had to carry out an additional task where they were asked to touch the thumb with the index finger of the non-dominant hand as fast as they could (maximum finger tapping speed). The above calculated performance rate in the sequential FT task was corrected with the maximum motor speed by subtracting the time between finger taps in the maximum motor speed task from the time between taps in the sequential task, then it was multiplied by the error rate of the sequential task.

#### Navon Global–Local Task

The Navon global–local task consisted of hierarchical stimuli of geometric shapes, often called Navon figures (Navon, 1977, see **Figure 1C**), presented on a computer screen. In a Navon figure, the lines of a larger, “global” shape are composed of much smaller, “local” figures, 10th the size of the large ones. In this version of the task, geometric shapes (circle, triangle, x, and square) were used instead of letters to rule out the effect of reading experience in younger subjects. The Navon global–local task was presented using a HP ProBook 450 G3 laptop computer with a 15.6 inch monitor, and was programmed with the OpenSesame 3.0 software (Mathôt et al., 2012). Participants were instructed to identify the shape, either at the global or the local level, depending on the color of the background, which was blue or yellow, respectively. Subjects were asked to provide button-press responses on the computer keyboard. Stimuli were organized into three blocks. The first and second blocks involved 24 randomized non-shifting trials (only blue/global or only yellow/local) and the third consisted of 48 quasi-random shifting trials, where 25 of the trials required a switch from local to global features or vice-versa. The order of non-shifting trials was alternated among subjects, half of the subjects started with the global set of the task followed by the local set and vice versa. Each block was preceded with instructions and a practice block with 6 stimuli for the non-shifting, and 12 stimuli for the shifting condition. Stimuli appeared for 600 ms, followed by a visual cue for the location of the responding keys, and the trial ended with a resting screen. Responses were allowed only after the stimulus ceased from the screen. Reaction time (RT) and precision were recorded. We expressed the ‘cost’ of switching by calculating the difference between the RT of the correct responses in the alternation trials (participants were required to switch rules between two consecutive stimuli) and the average RT of the correct responses in the repetition global and local trials (participants did not have to switch rules).

## Results

### Data Analysis

For descriptive statistical analysis IBM SPSS Version 21.0 (IBM Corp., Armonk, NY, USA) was used. Performance less than 60% correct on one of the conditions or outliers with greater values than 2 SDs at age and gender group-level per condition were excluded from the analysis, which amounted less than 10% of the sample. **Table 2** presents the descriptive statistics of the data.

**Table 2:** Descriptive statistics of the data.

Age group		CI			FT			Navon GL		
		Mean	*SD*	*N*	Mean	*SD*	*N*	Mean	*SD*	*N*
**10 years**	Male	0.94	0.10	8	0.45	0.17	10	1266.14	790.06	10
	Female	0.91	0.11	9	0.64	0.20	10	1160.65	298.55	9
**12 years**	Male	0.92	0.11	9	0.73	0.21	8	789.58	547.99	7
	Female	0.83	0.14	10	0.64	0.11	11	839.71	390.00	11
**14 years**	Male	0.83	0.14	8	0.77	0.14	8	696.94	320.90	8
	Female	0.76	0.08	10	0.89	0.31	10	672.60	459.58	9
**16 years**	Male	0.78	0.06	8	0.88	0.25	9	655.42	359.96	9
	Female	0.73	0.09	10	1.06	0.25	9	470.92	224.82	10
**18 years**	Male	0.70	0.08	10	0.81	0.27	9	582.70	266.97	10
	Female	0.73	0.10	9	0.88	0.34	10	529.07	220.04	10
**20 years**	Male	0.74	0.06	11	1.03	0.14	10	628.25	467.33	10
	Female	0.70	0.08	9	0.90	0.22	10	448.75	190.81	11

*In the CI task, D ranged between 1.1 and 0.6, where the easiest level was *D* = 1.1 followed by more difficult levels up to *D* = 0.6 (lower scores reflect better performance). In the FT task, corrected performance rate ranged between 0.114 and 1.519 (lower value indicates poorer performance). In the Navon global–local task, the results ranged between 83 and 3239 ms (lower scores indicate better performance).*

Multivariate ANOVA was used to analyze the relationship between each behavioral task, gender and age.

Higher levels of contour integration (CI) performance were associated with development [*F*(5,99) = 14.210, *p* < 0.001, $\eta_{p}^{2}$ = 0.418], and gender [*F*(1,99) = 5.296, *p* = 0.023, $\eta_{p}^{2}$ = 0.051], females demonstrating better performance. Gender × development interaction was not present [*F*(99,546) = 0.809, *p* = 0.546, $\eta_{p}^{2}$ = 0.039]. Simple contrasts test revealed 10 [*t*(35) = -6.272,35, *p* < 0.001, *r*^2^ = 0.727], 12 [*t*(37) = -4.844, *p* < 0.001, *r*^2^ = 0.623], and 14 [*r*(36) = -2.469, *p* = 0.016, *r*^2^ = 0.381] years old adolescents showing a significantly poorer performance than the 20 years old group (adults).

Higher levels of finger tapping (FT) performance were associated with age [*F*(5,102) = 10.326, *p* < 0.001, $\eta_{p}^{2}$ = 0.336], but there was no main effect of gender [*F*(1,102) = 1.723, *p* = 0.192, $\eta_{p}^{2}$ = 0.017], or gender × age interaction [*F*(5,102) = 1.777, *p* = 0.124, $\eta_{p}^{2}$ = 0.080]. Simple contrasts test indicated significant differences between 10 [*t*(35) = -5.875, *p* < 0.001, *r*^2^ = 0.705] and 12 [*t*(37) = -3.877, *p* < 0.001, *r*^2^ = 0.537] years olds versus adults.

Higher performance on the Navon global–local task was associated with age [*F*(5,102) = 7.794, *p* < 0.001, $\eta_{p}^{2}$ = 0.276], but there was no main gender effect [*F*(1,102) = 1.159, *p* = 0.284, $\eta_{p}^{2}$ = 0.011] or gender × age interaction [*F*(5,102) = 0.233, *p* = 0.947, $\eta_{p}^{2}$ = 0.011] evident. Simple contrasts test revealed, that 10 [*t*(38) = 5.22, *p* < 0.001, *r*^2^ = 0.646] and 12 [*t*(77) = 2.078, *p* = 0.04, *r*^2^ = 0.319] years olds preformed significantly worse, than adults (20 years).

### Sigmoid Curve Fitting

**FIGURE 2:**
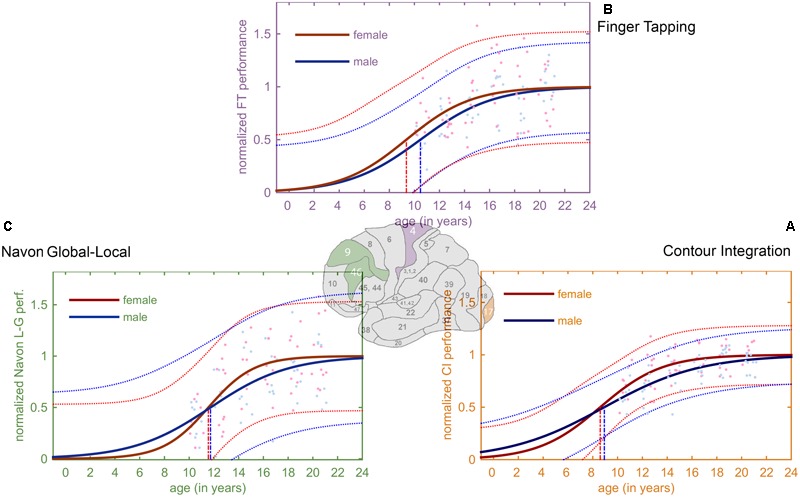
**Derived developmental trajectories for the three tasks.** The curves exhibit the characteristic ‘S’-shaped curve of a sigmoid. Red and blue stands for females and males, respectively. Lighter red and blue dots represent the female and male normalized data, and the lighter, dotted lines depict the 95% prediction bounds. **(A)** Fitted sigmoid curves for normalized CI performance data. **(B)** Fitted sigmoid curves for normalized FT performance **(C)** Fitted sigmoid curves for normalized Navon global–local task.

As a fitting method we used the Curve Fitting Toolbox of MatLab (2014b). The three data streams were transformed so that their zero value has a meaning of the entire function missing thus enabling us omitting one parameter (see **Figure 2**). We fitted data using the Curve Fitting Toolbox of MatLab (2014b) with the equation of

$$Predicted\;function\;level=0+\frac{Saturation level}{{1+e}^{\frac{\mathrm{inflexion}\;point-age}{\mathrm{development}\;\mathrm{speed}}}}$$

Different fittings were calculated for females and males and after the fitting process we transformed the data and the fitted curves in a way that different saturation levels of males and females were filtered out their respective saturation levels transformed to 1. Thus, we eliminated gender performance differences to concentrate purely on the developmental process dynamics of the two genders.

We also constrained the saturation level parameter between 0 and 2, the age at development deceleration parameter between 0 and 25 and development speed parameter between 0.5 and 25, note that age was measured in months before transformation. The descriptive details of the fitted models are presented in **Table 3**. A potential limitation of the interpretation of our data is that the model explains less than 50% variance. For further theoretical and technical details about the curve fitting see Supplementary Material.

**Table 3:** Details of the fitted sigmoid curves for females and males in the three tasks.

		Inflection point	95% CI lower bound	95% CI upper bound	90% saturation	Relative saturation	Relative acceleration	Model adjusted *R*^2^
**CI**	Female	8.6 years	6.8 years	10.4 years	14.1 years	0.99	1.28	36.14%
	Male	8.9 years	7.2 years	10.6 years	17.5 years	1.01	0.82	47.29%
**FT**	Female	9.4 years	7.0 years	11.7 years	15.9 years	0.90	1.38	18.89%
	Male	10.5 years	9.1 years	11.9 years	18.7 years	1.10	0.78	39.96%
**Navon GL**	Female	11.6 years	10.6 years	12.5 years	15.6 years	0.99	1.35	40.16%
	Male	11.7 years	9.8 years	13.6 years	18.6 years	1.01	0.79	25.08%

*Relative saturation is the relative measure of the jointly fitted model saturation. Relative acceleration is the relative measure of the theoretical mean of the two genders’ acceleration parameters.*

### Comparing Developmental Trajectories

**FIGURE 3:**
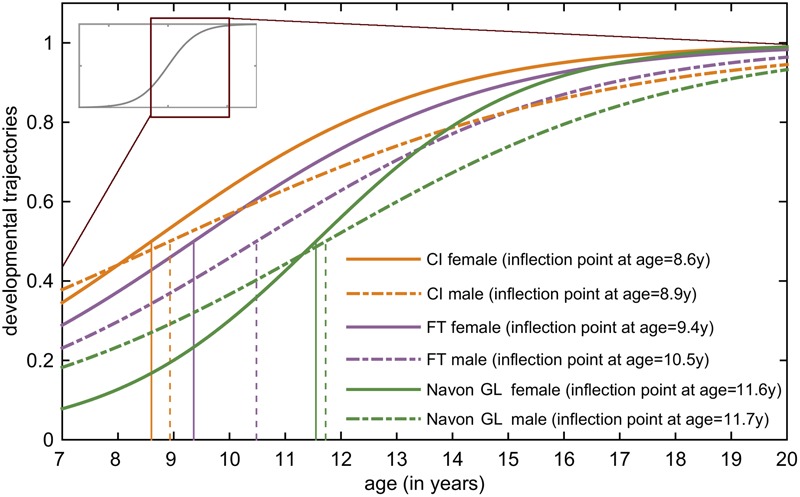
**Developmental trajectories of the tree tasks for females and males.** In this figure, the most relevant areas of the fitted sigmoid curves are highlighted: these are the steepest parts of the slopes between the lower and upper plateau, the so called inflection points. The vertical lines project the inflection points of the sigmoid curves to the *x-*axis, showing the age when the maximum acceleration occurs according to the model. The earliest inflection points occur in the CI task (girls: 8.6 years, boys: 8.9 years), followed by the FT task (girls: 9.4 years, boys: 10.5 years). The latest inflection points appear in the Navon global–local task (girls: 11.5 years, boys: 11.7 years).

**Figure 3** illustrates the developmental trajectories of the tree tasks (**Figures 2A–C**) for females and males, and the maximum acceleration timings of these functions. By analyzing the developmental trajectories of the three tasks, we found a posterior to anterior pattern in the emergence of the inflection points of the fitted sigmoid functions. Inflection points are at the earliest ages in the CI task (8.75 years on average), the FT task follows (9.95 years on average), and the last is the Navon global–local task (11.6 years on average).

Gender differences were also present in all tasks showing an earlier development for girls. A small gender difference occurred in the CI task (inflection at 8.6 years for girls, and 8.9 years for girls). In the FT task the difference was larger (9.4 years for girls, and 10.5 years for boys), and it was also relatively small in the Navon global–local task (11.5 years for girls, and 11.7 years for boys).

## Discussion

Our purpose was to investigate the similarities between behavioral development and brain maturation during childhood and adolescence. We focused on three specific functions related to distinct cortical areas. The behavioral paradigms were well-established paradigms with extensively studied neural correlates: (i) low-level visual spatial integration relying on the primary visual area was addressed by contour integration task; (ii) fine motor control function, mediated by the primary motor cortex in the precentral gyrus in the posterior frontal lobe, was investigated by a self-paced finger tapping task; and (iii) executive control, mediated by the dorsolateral prefrontal cortical area, was studied by the Navon global–local task. After obtaining data from 122 typically developing subjects, we applied fitted sigmoid curves on the normalized and corrected raw performance data of the three tasks for females and males. Sigmoid curves have a distinguished part called inflection point which signifies the point where development is the fastest and most prominent. Acknowledging the theoretical considerations and neural correlates provided by several researches for the three tasks we employed, we found a similar posterior to anterior pattern in the maturation of the task related functions. Emergence of the inflection points of the fitted sigmoid functions is earliest in the case of the CI task (female = 8.6 years; male = 8.9 years), the FT task follows (female = 9.4 years; male = 10.5 years) and the last is the Navon global–local task (female = 11.5 years; male = 11.7 years). Imaging studies also agree on the earliest maturation of the occipital pole (Sowell et al., 2003, 2004, 2007; Gogtay et al., 2004).

Developmental changes of the frontal lobe display an inhomogeneous timing; maturation shows roughly a posterior to anterior direction with an exception of the frontal pole that matures at about same time as posterior regions (Gogtay et al., 2004). Accordingly, developmental alterations start earliest in the precentral gyrus (primary motor cortex), and the prefrontal cortex matures last (Gogtay et al., 2004). The data obtained from the sigmoid models are consistent with the imaging findings revealing a posterior to anterior direction of frontal lobe maturation, sigmoid curves of FT task show earlier inflection points than curves related to Navon global–local task.

Brain maturation displays a gender specific timing and pattern during childhood and adolescence (for review see e.g., Lenroot and Giedd, 2010), girls mature earlier than boys. Our results also show gender difference in the timing of the changes in behavioral functions connected to M1 area, while there is only a slight difference between males and females in the visual and executive control task. In the fine motor task (FT), female and male developmental curves reach the inflection points by the age 9.4 and 10.5 showing 1.1 years difference between gender groups. Delayed developmental growth curves for males in the FT task could be related to later volume growth peaks for frontal GM in males (Giedd et al., 1997; Giedd and Rapoport, 2010) and earlier myelination for females (Benes et al., 1994). The earlier inflection points in females compared to males may reflect an influence of sex hormones on the maturation of these brain regions. In the Navon global–local task, the female and male developmental curves reach the inflection points by the age of 11.5 and 11.7, respectively. The lack of significant gender difference might be explained by the explained by the massive inhomogeneity of males’ performance in this task. In CI task, female and male developmental curves reach the inflection points by the age of 8.6 and 8.9, respectively. This is consistent with the findings of a recent structural MRI study (Koolschijn and Crone, 2013) where data show only moderate occipital volume gray matter loss after age of 8, indicating that the vast majority of structural maturational changes of this lobule have proceeded by this age. The volumetric loss in gray matter is likely associated with two simultaneous maturational processes: synaptic pruning, i.e., the elimination of unused synaptic connections (see e.g., Huttenlocher and Dabholkar, 1997); WM growth, i.e., myelination (see e.g., Benes et al., 1994; Courchesne et al., 2000; Gogtay et al., 2004). However, the study of Koolschijn and Crone (2013) reports temporal, parietal and frontal gray matter volume loss in early adolescent years which could be associated with more protracted brain maturation in these lobules. The occurrence of the maximum acceleration of maturation prior to puberty, and the lack of significant gender differences in the CI task might imply that the maturation of this visual function is less hormonally driven than the other two investigated functions.

We conclude that the posterior to anterior structural and functional maturational direction of the human brain could be grasped by behavioral paradigm addressing specific cortical areas. Clearly, further research with younger age groups is needed to verify our developmental trajectory predictions regarding the exact timing of the maximum accelerations of the investigated functions.

## Author Contributions

IK and PG contributed to the conception and design of the research, and wrote the paper. OF coordinated data acquisition and analysis. PS fostered data analysis and the interpretation of the results. AB participated in the analysis of the data. All authors discussed the results and implications and commented on the manuscript at all stages. All authors approved the manuscript and this submission.

## Conflict of Interest Statement

The authors declare that the research was conducted in the absence of any commercial or financial relationships that could be construed as a potential conflict of interest.
