# Supplementary material for: Posterior–Anterior Brain Maturation Reflected in Perceptual, Motor and Cognitive Performance
Source: Front Psychol. 2017 May 2;8:674. doi: 10.3389/fpsyg.2017.00674 (PMC5411422; doi:10.3389/fpsyg.2017.00674)
Supplement: Supplementary file 1 [file Data_Sheet_1.pdf]

Supplementary material

The use of linear regression for modelling developmental features is somewhat misleading, as most if not all developmental features are constrained between a minimum value, where the feature observed is in an undeveloped stage, and a maximum value, that can be considered as a fully developed stage. In our case the interval of development between the undeveloped and developed features are bigger than the variation that can be found in any single age cohort observed. Since our independent variable is age, the chosen modelling scheme must reflect some properties of development as a general process as a function of time. Development, as a process, starts from a baseline value that can be interpreted as the missing developmental feature, or as the basis on which the development occurs. This baseline capability then begins to increase due to the developmental process, and reaches a maximum acceleration (the inflection point of the curve). This signifies the point when the development is the fastest and most prominent. Then, the developmental process slows down due to the saturation of the biological capabilities and maturing program, and when reaches the developmental limit, it stays at a high level constantly. The best suited fitting curve that answers this purpose is the sigmoid curve. Several other studies used one or the other from this family of curves for human developmental modelling. A study by Jando and colleagues (2012) demonstrated the experience-dependence with the use of sigmoid fitting: preterm infants developed binocular vision, earlier, when age was adjusted to conception, but curves overlapped, when postnatal age was considered. Accelerated maturation of white matter was observed in young children with autistic spectrum disorder using log-sigmoid model-fitting (Ben Bashat et al., 2007). Dean and colleagues (2014) modeled healthy male myelination between 3 and 60 months applying a slightly modified version of the log-sigmoid curve. Note that a similar set of authors published a study where they modelled such data with a log curve (Deoni et al., 2012). However, a log curve is very similar to the sigmoid curve after correct parameterization of its inflection point,

and the log sigmoid curve has more theoretical basis, especially when interpreting data related to a function that more or less saturates to a level in early adulthood. Therefore, we limit the non-linear fitting to these types of curves. There are more considerations to further limit these curves and their parameters. Generally, the fitted curves have to reflect some acceptable developmental process: firstly, the flat baseline of the curves should occur at an interpretable distance from the biological beginning of life; secondly, for the same feature fitted for boys' and girls' data, the baseline should be at the same level as the theoretical null point of development. As for the first constraint, being too strict would be false, for the developmental process is much more complex than any sigmoid curve could suggest, and development might speed up, or slow down at different ages more than reflected in such a model. However, strictness is favourable in the case of the second feature because the dependent variable is common in the two genders.

As a fitting method we used the Curve Fitting Toolbox of MatLab (2014b). The six databases were transformed so that their zero value has a meaning of the entire developmental function baseline, thus enabling us omitting one parameter to vary freely (see Figure 2). We fitted data using the Curve Fitting Toolbox of MatLab (2014b) with the equation of

$$predictedFunctionLevel = 0 + \frac{saturationLevel}{1 + e^{\frac{inflexionPoint - age}{developmentSpeed}}}$$

Different fittings were calculated for females and males, and after the fitting process, we transformed the data and the fitted curves in a way that different saturation levels of males and females were filtered out, their respective saturation levels transformed to 1. Thus, we eliminated gender performance differences to concentrate purely on the developmental process dynamics of the two genders. This latter transformation does not affect the inflection points reported.

Note that using nonlinear models often enhances model accuracy. Considering linear regression as the "smart" baseline model, there are multiple choices from a range of fitting methods and

algorithms. Since there are multiple non-linear fitting methods available, trying multiple models, and finding one that is better than the “smart” baseline, is almost evident, that is, if not theoretically underpinned, raises a problem similar to inflated Error Type I. Therefore, we limited ourselves to a single non-linear model that is theoretically most suitable to fit our data, furthermore we fixed one parameter of the sigmoid curve so that it reflects a developmental starting point.

When compared to linear models, the sigmoid models perform better, in each case producing higher R^2 values on the datasets. Using F statistics, and Akaike Information Criterion (AIC) to compare the models with respect to the different degrees of freedom, we can still observe slightly better fitting with the sigmoid curves than by using linear regression, except for two cases (see Table S1). In general, the sigmoid curve fits better the data, even considering the shrinking in the degrees of freedom. More importantly, the linear model has no theoretical basis: the two parameters vary freely, with uninterpretable predictions for the populations before and after the cohorts are measured.

Table S1 Linear and Sigmoid model comparison

		**Unadjusted R^2**		**model parameters**			**F-statistics**		**AIC**
		**sigmoid**	**linear**	**df-sig.**	**df-lin.**	**N**	**F**	**p**	**d-AIC**
**Contour Integration**	female	0.373	0.319	54	55	57	4.66	0.035	2.72
	male	0.483	0.452	51	52	54	2.99	0.090	1.07
**Finger Tapping**	female	0.203	0.156	57	58	60	3.35	0.072	1.43
	male	0.411	0.400	51	52	54	0.94	0.337	-1.01
**Navon GL**	female	0.423	0.368	55	56	58	5.16	0.020	3.20
	male	0.282	0.272	46	47	49	0.70	0.417	-1.29
